# Clinical profile and demographic characteristics of moderate and severe hemophilia patients in a tertiary care hospital of Bangladesh

**DOI:** 10.1186/s13023-022-02413-7

**Published:** 2022-07-08

**Authors:** Mohammed Nadimul Islam, Akhil Ranjon Biswas, Humayra Nazneen, Nobendu Chowdhury, Mahbubul Alam, Jayanta Banik, Md. Kamrul Hassan, Abdullah Az Zubayer Khan, Najmul Karim, Mohammad Jahid Hasan, Md. Abdullah Saeed Khan

**Affiliations:** 1 National Institute of Laboratory Medicine and Referral Centre, Dhaka, Bangladesh; 2 Department of Haematology and BMT, Dhaka Medical College Hospital, Dhaka, Bangladesh; 3 Department of Haematology, Dhaka Medical College Hospital, Dhaka, Bangladesh; 4 Upazilla Health Complex, Golapgonj, Sylhet, Bangladesh; 5 Sheikh Russell National Gastroliver Institute and Hospital, Dhaka, Bangladesh; 6 250 Bed General Hospital, Kishoregonj, Bangladesh; 7 Department of Pathology, Shaheed Syed Nazrul Islam Medical College, Kishoreganj, Bangladesh; 8 Department of Haematology, National Institute of Laboratory Medicine and Referral Center, Dhaka, Bangladesh; 9 Medicine Department, Rangpur Medical College Hospital, Rangpur, Bangladesh; 10 Pi Research Consultancy Center, Dhaka, Bangladesh

**Keywords:** Hemophilia, Severity, Factor VIII, Factor IX, Bangladesh

## Abstract

**Background:**

Hemophilia is one of the commonest inherited bleeding disorders which may lead to chronic bleeding tendencies and life-long disabilities if not properly managed. Knowing the pattern of the disease aids in the prevention of disability and improvement of quality of life in hemophilia. However, there is a dearth of literature on the issue in Bangladesh. So, this study was designed to explore the frequency and site of spontaneous bleeding in moderate and severe hemophilia patients visiting in a tertiary level hospital.

**Methods:**

This descriptive cross-sectional study was conducted at the department of Hematology and Bone Marrow Transplantation (BMT) Center in Dhaka Medical College Hospital, Dhaka between February 2020 and August 2020. A total of 44 diagnosed cases of moderate to severe hemophilia were included in the study according to inclusion criteria. A detailed inquiry of history, thorough physical examination and relevant investigations were done and were recorded in case-record form. Informed written consent was taken from patients or their guardians where appropriate. All procedures were done according to Declaration of Helsinki. After entry and checking, data was analysed using SPSS version 26.

**Results:**

Out of 44 participants, 25 (56.8%) and 19 (43.2%) had moderate and severe hemophilia. Mean age of the study population was 21.31 (± 9.78) years with the majority aged between 11 and 20 years (45.5%). All sociodemographic features were similar across severity. Hemophilia A and B was found in 90.9% and 9.1%, respectively. However, all type B patients severe hemophilia making it statistically significantly different from type A (p = 0.029). The median age of first bleeding was 3.5 years and median age of first diagnosis was 5 years. Nevertheless, approximately 67.4% patients were diagnosed as a case of hemophilia at the time of their first diagnosis. The median spontaneous bleedings episodes among all patients was 32 (range: 0–97) which did not different significantly between severe and moderate patients. The most common affected (target) joint was knee joint (88.6%) followed by elbow joint (64%) among all patients. The knee joint was more commonly involved in severe than moderate disease.

**Conclusion:**

This study observed the variations in pattern and frequency of spontaneous bleeding in patients with hemophilia. Severe disease was more frequent in hemophilia B than A and knee joint was the most frequent site of bleeding. However, further extensive studies are recommended.

## Background

Hemophilia A (Classical) and B (Christmas disease) are congenital bleeding disorders caused by a deficiency or complete absence of coagulation factor VIII (FVIII) or factor IX (FIX), respectively. Hemophilia A and B constitutes 80% and 20% of all hemophilia cases [1]. It represents the large majority of hereditary clotting disorders, with hemophilia A and B occurring in approximately one per 5,000 and one per 30,000 male births, respectively [2]. However, approximately 30% cases occurs due to de novo mutations and do not have a family history [3]. Globally, 1,125,000 patients have hemophilia [4]. Out of them, 80% are in developing countries [3]. Bangladesh contributes a great portion to this patient burden. There are 1675 registered patients of hemophilia according to the ‘World Hemophilia Registry’. The prevalence of hemophilia in this country is 10 per million people [5], though the actual prevalence would be much higher as the survey system is not strong enough to include and monitor all the patients regularly.

Hemophilia patients are classified according to their residual endogenous FVIII/FIX concentrations. Subjects with factor levels < 1 IU/dl, 1–5 IU/dL and > 5 IU/dL have severe, moderate and mild hemophilia, respectively. About half of the patients with hemophilia have severe disease. But patients show heterogenous bleeding presentation even in severe disease [6]. The classification of hemophilia provides guidance to possible types and frequency of recurrence of bleeding. Patients with severe hemophilia might experience spontaneous bleeding, while patients with moderate form develop bleeding after mild to moderate injuries. Mild hemophilic patients may live years before diagnosis and presents with hemorrhage only after surgery or major injury [7].

Hemarthrosis is the hallmark of a severe form of hemophilia [8] and is the most frequency occurring complication [9]. It occurs either spontaneously or after trauma. In Bangladesh, more than four-fifth hemophilic children were found to have joint symptoms with knee joint arthropathy being the predominant [1]. However, some European studies found predominantly ankle joint involvement [10, 11]. Muscular bleeding occurs in 10–25% hemorrhagic episodes of severe hemophilia [7] and is regarded as one of the major cause of disability in this disease. About three-quarter patients with severe hemophilia experience hemorrhage in muscle in their life time. Iliopsoas haematoma is a life-threatening hemorrhage which might cause femoral nerve compression [12]. Another cause of morbidity and mortality in hemophilia is CNS bleeding [8]. Gastrointestinal bleeding occasionally occurs in hemophilia. It may occur spontaneously or secondary to common causes of gastrointestinal bleeding. Haematuria is another troublesome presentation of hemophilia complicating hydronephrosis or ureteral obstruction in some patients [7].

Exploration of frequency and sites of bleeding among hemophiliacs would help in the anticipation and early detection of hemophilia in previously undiagnosed or unsuspected cases.. Thereby, it allows taking timely precautions. Hence the current study was designed to describe the demographic and clinical features of moderate to severe hemophilia patients with particular emphasis to the sites and frequency of bleeding.

## Materials and methods

### Study design, place and participants

This cross-sectional study was conducted in the Department of Hematology and Bone Marrow Transplantation (BMT) Center, Dhaka Medical College Hospital, Dhaka, Bangladesh over a period of six months between February 2020 and August 2020. All consecutive patients known or suspected to have hemophilia attending in the out-patients unit of hematology department, irrespective of age, were approached. Informed written consent from the patients or their guardians where appropriate were taken before inclusion. Only moderate (factor level 0.01–0.05 IU/ml) and severe (factor level < 0.01 IU/ml) hemophilic patients were included. Patients with a diagnosis of hematological malignancy, post-traumatic bleeding, acute liver failure, chronic liver disease, or any other bleeding disorder were excluded. Finally, a total of 44 patients with moderate to severe hemophilia got enrolled in the study. None of the patients were in prophylactic treatment.

### Study measures and procedure

Data collection was done in a pretested case record form (CRF) through face-to-face interview, and from patients’ records. The CRF consisted of several parts seeking demographic features, clinical information and investigation profile. Demographic part included age, weight and height of the patients. Weight was measured using a bathroom scale (OSAKA digital weight machine). Height was measured using a standard measuring tape. Clinical information comprised of family history of hemophilia, age of the first bleed, site of first bleed, age of diagnosis, bleeding history of last two-year, age of first joint bleed, most affected joints (target joints) and other forms of bleeding manifestation with their frequency of occurrence, and a general assessment of function. Target joint was defined as the joint with special predilection to develop hemarthrosis. Joint/soft tissue swelling was defined as any visible swelling of joints or soft tissue complained by the patients or their guardian, and confirmed by clinical inspection. Similarly, muscle wasting was defined as visible loss of muscle bulk complained by the patients or their guardian, and confirmed on clinical inspection and palpation. Participant’s functional status was evaluated at four different levels-unrestricted activity, full school/work with some limitation, restricted school/work and restricted self-care. It was developed for a general assessment of the affected participant’s function based on the functional levels of FISH (Functional Independence Score in Hemophilia) [13]. Investigations mainly included complete blood count using a Hematology Analyzer (XN-350 Sysmex), prothrombin time (PT), activated partial thromboplastic time (APTT), quantitative assay of factor VIII and factor IX quantified in a fully automated Hemostasis Analyzer (Stago STA Compact Max), X-ray of the ffected joint using X-ray machine (GE High Frequency Digital X-Ray Machine*)*, and ultrasonogram (USG) of suspected group of muscles. USG of whole abdomen was done to identify any intraperitoneal or retroperitoneal collection of blood, if clinically suspected. In suspected cases of intracranial haemorrhage, a CT scan of brain was carried out to confirm and classify intracranial haemorrhage.

After enrolment of patients a detailed general and systemic examination was done to identify the current evidence of bleeding. All new cases were subjected to factors VIII and IX assay. In old cases, factor level was reconfirmed only in patients where it had previously been done within 24 h of receiving factor VIII/IX or blood products or in patients having no authentic document in support of their diagnosis.

### Ethical considerations

Formal ethical clearance for the study was taken from the ethical review committee of Dhaka Medical College Hospital (Memo no-ERC-DMC/ECC/2020/50, Date: 19.02.2020). Informed written consent were taken before enrolment from the patients or from the guardian where appropriate. Anonymity and confidentiality were maintained regarding participants and information obtained from them. All the procedures were done in accordance with the Declaration of Helsinki.

### Statistical analysis

Following data collection, the collected data were assessed for completeness, accuracy, and consistency before commencing analysis. Data analysis was carried out using SPSS version 26 (IBM Corp., Armonk, NY). Exploratory analysis was carried out to describe the study population where categorical variables were summarized using frequency tables and continuous variables were summarized using measures of central tendency and dispersion such as mean ± standard deviation (SD) and median (range). Qualitative or categorical variables were described as frequencies and proportions. Chi-square test and Fisher’s exact test were used to determine association between categorical variables. Normality of numerical variable were checked using Gaussian curve, Shapiro–Wilk test and Kolmogorov–Smirnov tests. Independent samples t test and Mann–Whitney U test were used to analyze quantitative data with normal and skewed distribution, respectively. A level of p-value < 0.05 was considered statistically significant.

## Results

**Fig. 1:**
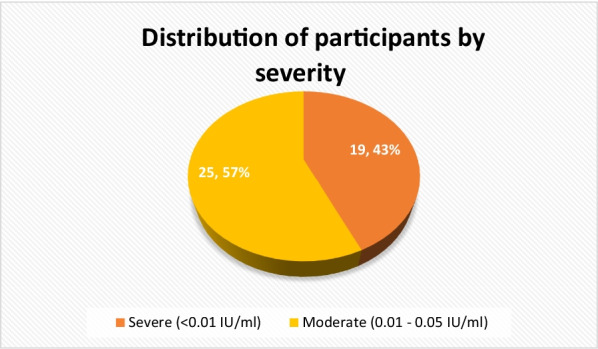
Distribution of participants by severity of hemophilia (n = 44)

Out of 44 participants 25 (56.8%) and 19 (43.2%) had moderate and severe hemophilia (Fig. 1).

The average age of the participants at the time of enrollment was 21.31 ± 9.78 years (SD) and majority respondents were aged between 11 and 20 years (45.5%). Maximum participants came from rural area (56.8), completed primary education (43.2%), were students (45.5%), and had a monthly family income of < 15,000 BDT (43.2%). All the socio-demographic features were similar across severity of hemophilia. The average BMI was 18.9 ± 2.3 kg/m^2^. Of all, 40 (90.9%) had hemophilia A and 4 had hemophilia type B (9.1%). All type B patients had severe hemophilia making the distribution statistically significantly different than hemophilia A (p = 0.029). Family history of hemophilia was present in 61.4% of the participants. The median age of first bleeding was 3.5 years and median age of first diagnosis was 5 years. Around 67.4% of the patients were diagnosed as a case of hemophilia at the time of their first bleeding. None of these differed significantly between severe and moderate hemophilia patients (Table 1).

**Table 1:** Sociodemographic characteristics and disease profile of the participants

Variable	Total	Severity of hemophilia		*p* value
		Severe n (%)	Moderate n (%)	
Total, n (%)	44	19 (43.2)	25 (56.8)	
Age (years), mean ± SD	21.3 ± 9.8	20.3 ± 9.8	22.0 ± 9.9	0.574
Age group (years)				
≤ 10	4 (9.1)	2 (10.5)	2 (8.0)	0.793
11–20	20 (45.5)	10 (52.6)	10 (40.0)	
21–30	12 (27.3)	4 (21.1)	8 (32.0)	
31–40	8 (18.2)	3 (15.8)	5 (20.0)	
Residence				
Rural	25 (56.8)	12 (63.2)	13 (52.0)	0.459
Urban	19 (43.2)	7 (36.8)	12 (48.0)	
Education				
Illiterate	3 (6.8)	3 (15.8)	0	0.148
Preschooler	2 (4.5)	1 (5.3)	1 (4.0)	
Primary	19 (43.2)	7 (36.8)	12 (48.0)	
SSC	11 (25.0)	5 (26.3)	6 (24.0)	
HSC	5 (11.4)	3 (15.8)	2 (8.0)	
Graduate and above	4	0	4 (16.0)	
Occupation				
Toddler	2 (4.5)	1 (5.3)	1 (4.0)	0.828
Student	20 (45.5)	9 (47.4)	11 (44.0)	
Job	5 (11.4)	1 (5.3)	4 (16.0)	
Business	8 (18.2)	4 (21.1)	4 (16.0)	
Farmer	4 (9.1)	1 (5.3)	3 (12.0)	
Unemployed/dependent	5 (11.4)	3 (15.8)	2 (8.0)	
Monthly family income (BDT)				
< 15,000	19 (43.2)	7 (36.8)	12 (48.0)	0.718
15,000–30,000	22 (50.0)	11 (57.9)	11 (44.0)	
> 30,000	3 (6.8)	1 (5.3)	2 (8.0)	
Body mass index, kg/m^2^	18.9 ± 2.3	18.7 ± 2.5	19.0 ± 2.1	0.636
Type of hemophilia				
Hemophilia A	40 (90.9)	15 (78.9)	25 (100.0)	0.029
Hemophilia B	4 (9.1)	4 (21.1)	0	
Family history				
Present	27 (61.4)	10 (52.6)	17 (68.0)	0.300
Absent	17 (38.6)	9 (47.4)	8 (32.0)	
Age of first bleeding (years), median (min–max)	3.5 (0.1–20.0)	2.0 (0.1–10.0)	4.0 (0.6–20.0)	0.429
Age at diagnosis (years), median (min–max)	5.0 (0.6–32.0)	5.5 (0.6–32.0)	5.0 (0.6–31.0)	0.834
Duration between first bleeding episode and diagnosis (years)				
None	29 (67.4)	11 (61.1)	18 (72.0)	0.721
< 10	8 (18.6)	4 (22.2)	4 (16.0)	
≥ 10	6 (14.0)	3 (16.7)	3 (12.0)	
Missing value	1			

*p* value determined by Chi-square test, Fisher’s Exact test and independent samples t test where appropriate

*SSC* Secondary School Certificate, *HSC* Higher Secondary School Certificate

Table 2 shows that all causes of bleeding were present in all patients. Knee was the most common target joint (70.5%) followed in second by elbow (15.9%). Joint swelling, muscle wasting, history of bleeding/ecchymoses from skin/other orifices, and soft tissue/muscle swelling were present in 97.7%, 97.7%, 72.7% and 75.0% patients respectively. Majority respondents could go to school or do work with some limitation (59.1%), 36.4% had restricted work/school going capacity, 2.3% could do restricted self-care and another 2.3% could do unrestricted activity. However, none of the presenting features were significantly different for severe hemophilia compared to moderate hemophilia.

**Table 2:** Distribution of the respondents by their clinical presentation and investigation (n = 44)

Clinical presentation	Total (n = 44)	Severity of hemophilia		*p* value
		Severe (n = 19)	Moderate (n = 25)	
Mode of bleeding				
Spontaneous	44 (100)	19 (100)	25 (100)	–
After minor trauma	44 (100)	19 (100)	25 (100)	–
After major trauma	44 (100)	19 (100)	25 (100)	–
Joint swelling				
Yes	43 (97.7)	18 (94.7)	25 (100)	0.432
No	1 (2.3)	1 (5.3)	0	
Target joint				
Knee	31 (70.5)	11 (57.9)	20 (80.0)	0.268
Elbow	7 (15.9)	4 (21.1)	3 (12.0)	
Ankle	2 (4.5)	1 (5.3)	1 (4.0)	
Shoulder	2 (4.5)	2 (10.5)	0	
Hip	1 (2.3)	0	1 (4.0)	
None	1 (2.3)	1 (5.3)	0	
Soft tissue/muscle swelling				
Yes	32 (72.7)	15 (78.9)	17 (68.0)	0.419
No	12 (27.3)	4 (21.1)	8 (32.0)	
Muscle wasting				
Yes	43 (97.7)	18 (94.7)	25 (100.0)	0.432
No	1 (2.3)	1 (5.3)	0	
Bleeding/ecchymosis from skin/other orifices				
Yes	33 (75.0)	17 (89.5)	16 (64.0)	0.081
No	11 (25.0)	2 (10.5)	9 (36.0)	
Functional status				
Unrestricted activity	1 (2.3)	1 (5.3)	0	0.416
Full school/work with some limitation	26 (59.1)	10 (52.6)	16 (64.0)	
Restricted school/work	16 (36.4)	7 (36.8)	9 (36.0)	
Restricted self-care	1 (2.3)	1 (5.3)	0	
Investigation				
APTT (s)	89.30 (49.4–120.0)	88.65 (72.0–98.0)	89.4 (49.4–120.0)	0.951
X-ray of the affected joint				
Joint changes present	40 (93.0)	17 (89.5)	23 (95.8)	0.416
No change	3 (7.0)	2 (10.5)	1 (4.2)	
Missing value	1			
Ultrasonogram of suspected muscle group				
Normal	14 (34.1)	5 (29.4)	9 (37.5)	0.591
Muscle Hematoma	27 (65.9)	12 (70.6)	15 (62.5)	
Missing value	3			
Ultrasonogram of whole abdomen				
Normal	31 (72.1)	14 (73.7)	17 (70.8)	0.836
Retroperitoneal hemorrhage	12 (27.9)	5 (26.3)	7 (29.2)	
Missing value	1			

Data expressed as n(%) and median(min-max) where appropriate

*p* value was determined by Fisher’s Exact, Chi-square test, and Mann–Whitney U test where appropriate

*APTT* Activated Partial Thromboplastin Time

Overall, 93.0% showed changes of the affected joint in X-ray, 65.9% had ultrasonographic features of muscle hematoma and 27.9% had retroperitoneal hemorrhage at USG of whole abdomen (Table 2).

**Fig. 2:**
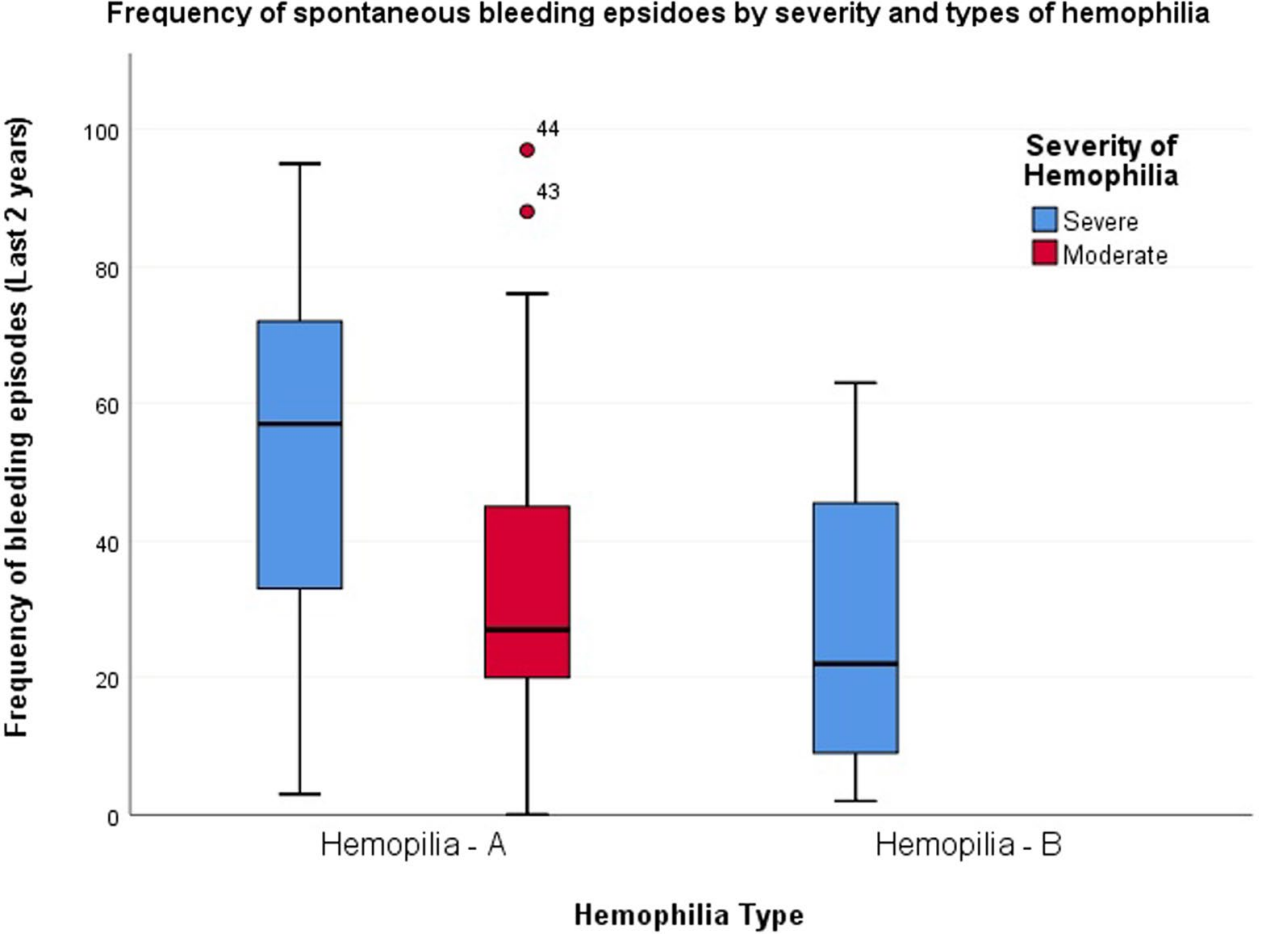
Frequency of spontaneous bleeding episodes by hemophilia type and severity (n = 44)

Table 3 and Fig. 2 show the site and frequency of spontaneous bleeding in relation to severity among hemophilia patients included in the study. The median spontaneous bleedings episodes during last two years were 49, 27 and 32 for severe, moderate and total hemophiliac patients, respectively, with a range from 0 to 97 events among all. The most common affected joint was knee joint (88.6%) followed by ankle and elbow joint (64% both). The median number of episodes of joint hemorrhage was also the highest for knee joints (12, range: 1–40). This was followed by elbow joints where the number of episodes ranged from 1 to 32 with a median of 9. Knee joint bleeding was more common among severe hemophiliacs and ankle, elbow and shoulder joint bleeding was more common among moderate hemophiliacs. However, the difference was not statistically significant. The most common site of muscle hemorrhage was thigh muscle which was relatively high among moderate hemophiliacs than severe hemophiliacs in terms of frequency, number of episodes and range. Again, the difference was not statistically significant. Gum bleeding was found in nearly half of both types of patients, bleeding episodes being higher among severe group. Melaena was present in 25% patients. Distribution was similar in both moderate and severe groups. Genitourinary bleeding was present in both groups of patients with a nearly equal frequency. No evidence of retinal bleeds or intracranial hemorrhage was noted. Paradoxically, only number of bruise and ecchymosis events were significantly higher among moderate than that of severe hemophilia patients (median [range] 1.5 [@@1–6] and 3.5 [@@2–12] respectively for moderate and severe hemophilia patients, p = 0.016).

**Table 3:** Site and frequency of spontaneous hemorrhage by severity of hemophilia (n = 44)

Variables	Proportion of Events			*p* value	Number of Events			*p* value
	Total (n = 44) n (%)	Severe (n = 19) n (%)	Moderate (n = 25) n (%)		Total (n = 44) (min–max)	Severe (n = 19) (min–max)	Moderate (n = 25) (min–max)	
Spontaneous bleeding (last 2 years)					32 (0–97)	49 (2–95)	27 (0–97)	0.157
Bruises and ecchymosis	18 (40.9)	10 (52.6)	8 (32.0)	0.168	3 (1–12)	1.5 (1–6)	3.5 (2–12)	**0.016**
*Joint hemorrhage*								
Knee	39 (88.6)	16 (94.2)	23 (92.0)	0.638	12 (1–40)	10 (2–32)	12 (1–40)	0.944
Ankle	30 (68.2)	14 (73.7)	16 (64.0)	0.495	3.5 (1–20)	4 (1–20)	3 (1–20)	0.951
Elbow	30 (68.2)	14 (73.7)	16 (64.0)	0.495	9 (1–32)	11 (2–32)	8.5 (1–24)	0.257
Wrist joint	12 (27.9)	7 (36.8)	5 (20.8)	0.245	3 (1–10)	3 (1–8)	3 (1–10)	0.876
Hip joint	13 (30.2)	7 (36.8)	6 (25.0)	0.401	2 (1–12.0)	1.5 (1–4)	2 (2–12)	0.394
Shoulders	18 (41.9)	6 (31.6)	12 (50.0)	0.224	2 (1–40)	9.5 (1–40)	2 (1–10)	0.125
*Muscle hemorrhage*								
Iliopsoas	12 (27.9)	6 (33.3)	6 (24.0)	0.712	1 (1–10)	1.5 (1–10)	1 (1–4)	0.589
Buttock	9 (20.9)	3 (16.7)	6 (24.0)	0.361	2 (1–6)	2 (2)	1.5 (1–6)	0.714
Calf muscle	16 (37.2)	7 (38.9)	9 (36.0)	0.847	1.5 (1–10)	2 (1–10)	1 (1–10)	0.837
Thigh	22 (50.0)	10 (52.6)	12 (48.0)	0.761	2 (1–12)	2 (1–8)	2 (1–12)	0.923
Flexor muscles of forearm	11 (25.0)	4 (21.1)	7 (28.0)	0.731	2 (1–10)	5 (1–10)	2 (1–6)	0.412
Scalp	1 (2.3)	–	1 (4.0)	1.000	1	–	1	–
Facial	4 (9.1)	2 (10.5)	2 (8.0)	1.000	1 (1–2)	1 (1)	1.5 (1–2)	1.000
*Mucosal bleeding*								
Gum bleeding	22 (50.0)	11 (57.9)	11 (44.0)	0.543	1 (1–10)	4 (1–20)	1 (1–20)	0.243
Epistaxis	6 (13.6)	3 (15.8)	3 (12.0)	1.000	1 (1–10)	1 (1–10)	1 (1–2)	1.000
*Gastrointestinal bleeding*								
Hematemesis	2 (4.5)	1 (5.3)	1 (4.0)	1.000	2 (1–3)	3	1	1.000
Melaena	11 (25.0)	5 (26.3)	6 (24.0)	1.000	2 (1–7)	4 (1–7)	1 (1–2)	0.082
Oro-pharyngeal	6 (13.6)	2 (10.5)	4 (16.0)	0.684	3 (1–4)	2.5 (1–4)	3 (1–4)	1.000
Retroperitoneal	12 (27.3)	5 (26.3)	7 (28.0)	0.901	1 (1–4)	3 (2–4)	1 (1–2)	0.095
Hemoptysis	3 (6.8)	3 (15.8)	–	0.073	2 (1–4)	2 (1–4)	–	–
*Ocular bleeding*								
Subconjunctival hemorrhage	5 (11.4)	3 (15.8)	2 (8.0)	0.638	1 (1–2)	1 (1–2)	1 (1)	0.800
Vitreous hemorrhage	1 (2.3)	–	1 (4.2)	1.000	–	–	–	–
Genito-urinary bleeding	16 (36.4)	9 (47.4)	7 (28.0)	0.220	1 (1–5)	1 (1–5)	1 (1–2)	0.470

*p* value was determined by Fisher’s exact test, Chi-square test and Mann–Whitney U test where appropriate; Significant* p* value was shown in bold

## Discussion

Hemophilia A and B are X-linked genetic diseases caused by mutation in the factor VIII and factor IX genes leading to coagulation disorders [2], the clinical form of which is identical [14]. They usually present as bleeding after minor insignificant trauma or as a spontaneous hemorrhage [15]. Based on the plasma levels of factors hemophilia patient may develop mild, moderate and severe form of the disease. Hemophilic patients develop various forms of bleeding manifestations and may present at various age depending on severity. But bleeding into a joint space, or hemarthrosis, is the hallmark of hemophilia [8]. Large joints are typically involved in various frequency. This study describes the sites and frequency of bleeding episodes among moderate and severe hemophilia along with sociodemographic profile of patients in Bangladesh.

A total of 44 hemophilia patients were included who had an average age of 21.31 ± 9.78 years (± SD) and a median age of 20 years at inclusion. Maximum patients were aged between 11 to 20 years (45.5%). Similar to our study Singh et al. also found majority (26.85%) patients between 11 to 20 years [16]. John et al. found a median age of 22 years among their participants which is also similar to our study [17]. Being a genetic disease majority of the patients suffer their first episode of bleeding at early age, but often they are late to diagnose their problem. We found a median age of first bleeding 3.5 years but the median age at diagnosis was 5 years. Similar picture was recorded by John and colleagues who reported a median age of first bleeding 1.5 years and median age at diagnosis 3 years [17].

Only moderate and severe hemophilia patients were included in this study—56.8% patient had moderate and 43.2% had severe disease. Contrary to findings from other studies [3, 16] we found a lower proportion of severe disease among our participants. This could be a random finding among the small sample in our study.

Most of the patients hailed from rural areas (56.8%) which might be a reflection of the general rural–urban distribution of the population in the Bangladesh. According to The World Bank estimates by 2019 Bangladesh had 62.9% population living in rural areas [18]. As majority of the participants in our study were young students (43.5%) we found majority having primary education (43.2%) followed by 25% having Secondary School Certificate (SSC). Most of the people came from middle socio-economic category followed by lower socio-economic category. This certainly echoes the public health facility utilization pattern by the people of Bangladesh as described by Mannan [19]. People from lower economic strata usually take services from public health facility and the present study was conducted in the largest government-run facility the country.

We found respectively 90.9 and 9.1% hemophilia A and B patients. This corresponds with the proportion of inherited disorders associated with bleeding where hemophilia A is the most common disorder (72.3%) and followed by hemophilia B (11.5%). An epidemiological study conducted on hemophilia patients in Iraq showed that 72.9 and 24.8% patients were affected by hemophilia A and B respectively [20]. While a hospital-based study in North India found approximately 94% and 6% cases of hemophilia A and B respectively. This indicate that hemophilia B might present less frequently than hemophilia A causing less pursual of hospital services, which is also supported by Franchini and Mannucci [21].

Among our patients, nearly one-third had a delayed diagnosis, and for 14% patients the delay was ≥ 10 years. This indicates there is still a gap in the detection and management of hemophilia cases. The diagnosis is often delayed if the disease is of mild or moderate severity, because the bleeding tendency is manifested at an older age [14, 22]. Moreover, spontaneous bleeding is uncommon in mild cases, and recurrent joint bleed may be present in up to 25% of moderate cases [22] leading to delayed suspicion. Minhas and Giangrande pointed out several important observations for the delay in the diagnosis of hemophilia. According to them failure among physicians to recognize the disease at their presentation is the primary reason for the delay [19]. Diagnostic confusion arises when patient present to the emergency department after accident with bruises or ecchymoses instead of hemarthrosis. However, in countries where circumcision is practiced and preferred at an early age, the first bleeding manifestation may occur during circumcision and hemophilia could be suspected and diagnosed. For instance, Qasim, Asif and Hasan in a study [23] conducted in Pakistan noted that prolonged bleeding during circumcision led to diagnosis of haemophilia in as many as 47.1% of their participants. But, lack of facilities to measure factor VIII, IX, von Williband factor and factor VIII inhibitor levels might often hamper diagnostic evaluation in suspected cases in low resource settings [24].

Every participant in our study had at least one episode of spontaneous bleeding, and bleeding from minor and major trauma. The most common target joint of spontaneous bleeding was knee joint (70.5%) which was disproportionately higher than other target sites including elbow, ankle, shoulder, and hip. Joint swelling was present in nearly all patients. But three-quarter patients had any episodes of soft tissue/muscle swelling, and skin bleeding. These findings are higher than that of Mishra and colleagues who noted knee as the target joint in 57.1% participants and joint swelling in 76.6% patients [25]. Nevertheless, joint bleed or hemarthrosis is commonly the first presentation as John and colleagues found [17] and knee is the most common affected joint as Singh et al. [19] as well as Payel and colleagues [3] noted. We noted that 36.4% patients had restricted schooling or work and one patient were restricted to self-care and 59.1% had some limitation constituting 97.7% patients having at least some compromise in the joint movement due to hemophilic arthropathy.

Clinically severe disease presents with frequent spontaneous bleeding with a high frequency of joint crippling, whereas joint crippling is less common among moderate hemophilia patients [14]. We found that knee is the most common (88.6%) bleeding site followed by ankle and elbow (68.2%) and this was true for both severe and moderate disease. Median bleeding episode was high for elbow in severe disease and knee for moderate disease where bleeding episodes ranged from 1 to 40 in case of knee and 1 to 32 in case of elbow. Thigh was the most common site to have muscle hemorrhage naturally as it has the most muscle bulk and is subjected to repeated movements. Gum bleeding was present in fifty percent of the participants and was more common among severe hemophiliacs (median episode 4 vs 1 in cases of moderate disease). A considerable number of patients also had genitourinary bleeding (36.4% of total). Other forms of bleeding were less common but nonetheless present. However, we found that our pattern corresponds with the conventional knowledge regarding hemophilia [14] where large synovial joints are most commonly and frequently affected by spontaneous bleeding. The only unintuitive finding was a significantly higher median frequency of bruises and ecchymosis in moderate hemophilia patients compared to severe ones. However, the proportion of participants who had at least one event of bruise and/or ecchymosis was relatively higher in severe cases. It indicates that in the preceding two years participants of our study with moderate hemophilia had a higher number of bruising/ecchymosis events. One possibility of such an aberrant finding is the underreporting of such events by the participants with severe hemophilia. As participants were asked to recall such events from their memory the possibility of recall bias could not be excluded. Probably small bruises passed unnoticed among the severe hemophiliacs on the background of joint bleeds. Because, a careful survey of the frequency of joints bleeds among the participants indicate a non-significantly higher median number of bleeding episodes among severe cases. The major concern for hemophilia is intracranial bleeding, and sub-pharyngeal hematoma causing blockage of respiratory airway which was not found in our study.

None of the patients in our study were on prophylactic treatment. Hence a comparison of bleeding patterns between patients undergoing on-demand treatment and prophylactic treatment was not possible. However, previous studies conducted in experimental [26] and real-world settings [27] suggest that prophylactic treatment are effective in reducing bleeding frequency among hemophilia patients. So further studies exploring effectiveness of prophylactic factor replacement in hemophilia patients could be explored in the country.

In the light of our findings, and because of the fact that not all hemophilia patients and their families are enlisted in the registry, any event of sudden onset joint swelling, particularly of knee and elbow joint, in previously undiagnosed or unsuspected cases of hemophilia should be considered for exclusion of hemarthrosis and consequently of hemophilia as a priority. People with suspected thigh muscle pain and swelling should also be explored for possible presence of hemophilia.

Hemophilia has moved from a neglected and often fatal hereditary hemorrhagic disorder to that of a defined group of well-characterized molecular entities over the last three decades [28]. Developments in biotechnology and genetics have revolutionized the treatment of hemophilia by making available factors that are defective in this group of patients. Hence, an early diagnosis of hemophilia patients could be life-saving for many people as well as reduce the disability associated with the disorder. This study sorted the frequency of different forms of bleeding among severe and moderate hemophilia patients, thereby giving aid to developing a diagnostic protocol to decrease the diagnostic delay in nearly one-third of the patients.

Our study was limited in that it was conducted in a single center and only moderate or severe patients visiting the facility were included. Thus, we could study a small sample of hemophilic population. Moreover, inhibitor screening could not be done due lack of facility. However, the strength of our study is that a few studies reported a detailed history of sites of bleeding with their frequency of bleeding episodes among hemophilia patients in Bangladesh.

## Conclusion

In this study, more than half of the study population had moderate hemophilia. Severity was high among hemophilia B patients compared to hemophilia A. The most common bleeding site was knee and about three-quarter patients experienced episodes of soft tissue/muscle swelling, and skin bleeding. Median frequency of spontaneous bleeding among patients in last two years was 32 and was statistically similar in relation to severity. Based on the findings of this research it is recommended that clinicians should exclude hemarthrosis in previously unsuspected and undiagnosed hemophilia patients on the event of sudden onset joint swelling, particularly in knee and elbow joints.

## Data Availability

Please contact author for data requests.
